# Locus coeruleus integrity correlates with plasma soluble Axl levels in Alzheimer's disease patients

**DOI:** 10.1002/alz.70434

**Published:** 2025-07-01

**Authors:** Alessandro Galgani, Arnaud Mary, Francesco Lombardo, Nicola Martini, Marco Scotto, Gloria Tognoni, Gabriele Siciliano, Roberto Ceravolo, Filippo S. Giorgi, Michael T. Heneka

**Affiliations:** ^1^ Department of Translational Research and of New Surgical and Medical Technologies University of Pisa Pisa Italy; ^2^ Luxembourg Centre for Systems Biomedicine University of Luxembourg Belvaux Luxembourg; ^3^ Department of Radiology, Fondazione Toscana “G. Monasterio” Pisa Italy; ^4^ Istituto Italiano di Tecnologia Genoa Italy; ^5^ Department of Clinical and Experimental Medicine University of Pisa Pisa Italy; ^6^ I.R.C.C.S. Stella Maris, Calambrone Pisa Italy; ^7^ Division of Infectious Diseases and Immunology University of Massachusetts Medical School Worcester Massachusetts USA

**Keywords:** Alzheimer's disease, blood‐based biomarkers, locus coeruleus, neuroinflammation, noradrenaline

## Abstract

**INTRODUCTION:**

Locus coeruleus (LC) is one of the earliest structures altered in Alzheimer's disease (AD). Inflammation is also now considered critical in AD pathology, early stage included. However, no association between LC degeneration and the peripheral inflammation has been reported yet.

**METHODS:**

A cohort of 102 patients was studied for which both magnetic resonance imaging (MRI) scans and blood samples were available. LC integrity was assessed by MRI, and plasma soluble TAMs (Tyro3, Axl, and MerTK) receptor levels were measured by enzyme‐linked immunosorbent assay (ELISA).

**RESULTS:**

We found that plasma levels of the soluble TAMs receptor Axl were correlated with LC rostral degeneration in the whole cohort (*p* = 0.007), as well as in the AD+ group (*p* = 0.017), but not in the AD– group.

**DISCUSSION:**

These results uncover a new relationship between peripheric markers of inflammation and central early AD neurodegeneration.

**Highlights:**

- In Alzheimer's disease, no link between locus coeruleus degeneration and microglial activation was reported.
- Plasma Axl, Tyro3, and MerTK levels and locus coeruleus integrity were assessed in Alzheimer's disease patients.
- Locus coeruleus integrity positively correlates with plasma AXL, linked to microglia activation.
- Axl–noradrenergic signaling interplay deserves further larger longitudinal studies.

## 1 INTRODUCTION

The locus coeruleus (LC) is the primary source of noradrenaline (NA) for the central nervous system (CNS), projecting to nearly all major brain regions.^1^, ^2^ According to postmortem assays, the LC is one of the earliest brain structures to be involved in Alzheimer's disease (AD),^3^ with development of neurofibrillary tangles and existence of neuronal degeneration already in the prodromal stages of the disorder.^4^, ^5^, ^6^ Through magnetic resonance imaging (MRI), in vivo human studies confirmed this evidence, associating LC loss of integrity with amyloid beta (Aβ) deposition, tau cortical burden, and cognitive decline.^7^, ^8^, ^9^

At the same time, experimental preclinical data suggest that the loss of LC‐NA innervation might contribute to AD pathogenesis by exacerbating the neuroinflammatory response to Aβ.^10^, ^11^, ^12^ LC lesion in AD animal models have been shown to worsen inflammatory alterations,^13^, ^14^ disrupt microglial clearance activity,^15^, ^16^ and increase of Aβ accumulation.^13^, ^16^, ^17^ Longitudinal LC‐MRI studies indirectly support these preclinical data; elderly people with lower LC‐MRI signal were more likely to have memory decline at follow‐up,^18^ and individuals with mild cognitive impairment (MCI) who had more severe LC disruption had a higher risk of progressing to full dementia.^9^ However, to our knowledge, no clinical study has specifically explored in vivo the possible association between LC degeneration and AD‐related neuroinflammatory activity.

For the purpose of this study, we focused on analyzing a possible correlation with soluble TAM (Tyro3, Axl, and MerTK) receptor plasmatic levels. TAMs are part of the receptor tyrosine kinases family, which not only modulates cell migration, proliferation, and survival, but also reduces inflammation.^19^ These cell membrane receptors can undergo a shedding process, producing a soluble extracellular form that can be detected in cerebrospinal fluid (CSF) and blood.^19^ In the ADNI cohort, CSF Axl was one of the most significant proteins with an effect on longitudinal CSF Aβ_1‐42_, in subjects without any signs of Aβ pathology at baseline,^20^ and serum Axl was one of the 12 biomarkers associated with MCI.^21^ In the Swedish BioFINDER‐2 longitudinal cohort, higher baseline levels of CSF Axl and MerTK, in nondemented individuals at risk for AD (based on positive amyloid or tau imagery), predicted future slower tau accumulation and cognitive decline.^22^ In the DELCODE cohort, higher CSF levels of sAxl and sTyro3 correlated with larger brain structure and stable cognitive outcome at follow‐up,^23^, ^24^ but conversely, higher serum sAxl levels were related to lower structural integrity in Braak I, and lower cognitive performance at baseline.^25^

In this study, we explore the possible association between LC‐MRI signal and plasmatic levels of TAM receptors in a cohort of elderly subjects, both cognitively intact and with AD. Our aim is to provide in vivo evidence of the pathogenic role of LC‐NA system disruption in AD, potentially mediated by the loss of its modulatory effect on the neuroinflammatory response.

**RESEARCH IN CONTEXT**

1. **Systematic review**: We performed a PubMed search on the role of the locus coeruleus (LC) in modulating neuroinflammation and microglial function, both under physiological conditions and in Alzheimer's disease (AD). Preclinical literature consistently supports an anti‐inflammatory role of LC‐derived noradrenaline, primarily through modulation of microglial activity. Soluble TAM (Tyro3, Axl, and MerTK) receptors have emerged as potential biomarkers of microglial activation and neuroinflammation and have been investigated previously across the AD continuum. However, a possible association between LC degeneration and neuroinflammatory biomarkers has not yet been explored in vivo.
2. **Interpretation**: Our findings provide the first in vivo evidence in patients with AD of a possible association between LC degeneration and microglial activation, offering clinical support for preclinical data.
3. **Future directions**: The role of the LC in modulating neuroinflammation in AD warrants further investigation, as it may represent a promising target for disease‐modifying interventions.

## 2 MATERIALS AND METHODS

The study was conducted at the neurology clinic of the Pisa University Hospital, and the MRI scans were performed at Fondazione “G. Monasterio”‐CNR (Consiglio Nazionale delle Ricerche). The measurements of plasma biomarkers were performed at the Luxembourg Centre for Systems Biomedicine, University of Luxembourg.

### 2.1 Patient inclusion, blood sampling, and LC imaging

Patients fulfilling the diagnostic criteria for clinical AD dementia (ADD)^26^ and amnestic MCI^27^ were recruited consecutively together with age‐matched healthy controls (HC). All recruited participants were submitted to neurological evaluation, and detailed neuropsychological testing was administered either to assess the cognitive impairment severity in MCI and ADD or to confirm the preservation of cognition in HC (the full description of neuropsychological tests used can be found in Galgani et al.^9^). Global cognition was evaluated through Mini Mental State Examination (MMSE),^28^ whereas the Clinical Dementia Rating (CDR) scale^29^ and Neuropsychiatric Inventory (NPI)^30^ were used for scoring global functioning and behavioral symptoms, respectively. Subjects with major cardiovascular, metabolic or inflammatory disorders, psychiatric comorbidities, or severe chronic ischemic encephalopathy were excluded from the study.^9^

Blood withdrawal was performed via venipuncture within 1 week after the neuropsychological evaluation, consistently between 8:30 and 9:30 a.m. to minimize the risk of circadian physiological oscillations. Participants were asked to fast and to abstain from alcohol consumption (for 1 day before withdrawal) and from heavy physical activity (for 3 days before withdrawal). Anonymized blood samples were collected in ethylenediaminetetraacetic acid (EDTA) tubes, centrifuged at 3000 rpm for 10 min at 4°C immediately after collection, and then divided into 500 µL aliquots and stored at –80°C until biochemical assays. All subjects were submitted to high‐field 3T Brain MRI scan within 1 month from the neurological evaluation (MR unit: Excite HDx, General Electric with an eight‐channel phased‐array head coil). LC imaging was performed using a standardized template‐based approach (see Supplementary Methods), already described in Galgani et al.^9^ The LC Contrast‐Ratio (LC_CR_) was calculated for each participant for the entire LC (left and right averaged), and its rostral and caudal parts.

### 2.2 p‐Tau 217 plasma level measurement

Samples were thawed at room temperature and the tubes were vortexed for 5–10 s. Phosphorylated tau‐217 (p‐tau217) plasma levels were quantified in four separated runs after manually diluting the samples 1.5 times with Sample Diluent 1 (Fujirebio #292617). Plasma samples were poured into Hitachi sample cups (Fujirebio #80351) and placed in the Lumipulse G600II analyzer (Fujirebio #703380). Levels of p‐tau217 were measured automatically by the analyzer, via the chemiluminescent enzyme immunoassay technology, using the corresponding Lumipulse G immunoreaction cartridges (Fujirebio #81472), after controlling the cartridges by using the manufacturer's corresponding calibrators (Fujirebio #81471) and controls (Fujirebio #81473), following manufacturer's instructions.

### 2.3 Enzyme‑linked immunosorbent assay (ELISA) for biomarker measurements

Soluble TAM receptors plasma levels were quantified using the human soluble Tyro3 (R&D systems Cat#DY859), human soluble Axl (R&D systems Cat#DY154), and human soluble MerTK (R&D systems Cat#DY6488) DuoSet ELISA kits. Plasma samples were diluted two times before quantifying the TAM receptors levels. ELISA was carried out according to the manufacturer's instructions, and the optical density was read at 450, and 540 nm for correction, using a microplate reader (SpectraMax iD3, Molecular Device).

### 2.4 Statistical workflow

Participants were further stratified into AD+ and AD− groups based on their plasmatic p‐tau217 levels (cutoff: 0.37 pg/mL^31^), reflecting the likelihood of underlying AD pathology. For the current analysis, only HC individuals classified as AD− and AD+ patients were included.

After excluding outliers exceeding the Tukey outer fence, the normality of variables was assessed using the Shapiro–Wilk test. Because LC parameters followed a non‐Gaussian distribution, nonparametric tests were applied for comparisons. Spearman's correlation test was conducted to explore associations between variables.

All statistical analyses involving multiple comparisons were adjusted using false discovery rate (FDR) correction to minimize type I error. The significance level was set at *p* < 0.05. Statistical analyses were performed using SPSS version 25 (RRID:SCR_016479), and charts were generated with Prism GraphPad 9.0 (RRID:SCR_002798).

## 3 RESULTS

### 3.1 Description of the included population

One hundred thirty‐three patients were initially recruited for the study (41 HC, 63 MCI, and 29 ADD). Thirty‐one participants were excluded from the analysis due to a mismatch between their clinical and pathological classifications or because they were identified as outliers. The final population included 36 AD‐HC, 42 AD+ MCI, and 24 AD+ ADD. Diagnostic groups were balanced for sex and age, whereas, as expected,^32^ a higher frequency of the apolipoprotein E (*APOE*) ε4 risk factor was found in AD+ patients. A detailed description is reported in Table 1.

**TABLE 1:** Demographic, clinical, and genetic description of study population.

		AD‐ (HC)	AD+	MCI	ADD	AD–/AD+ (*p‐value*)	HC/MCI/ADD (*p‐value*)
	*N*	36	66	42	24	–	–
Sex	Male %	39%	30%	29%	33%	0.379	0.929
Age	Mean ± SD	71.94 ± 4.89	72.52 ± 4.74	72.38 ± 4.27	72.75 ± 5.57	0.661	0.693
*APOE* status	E4%	11%	32%	36%	25%	0.020*	0.042*, ^a^
p‐Tau 217	Mean ± SD	0.143 ± 0.060	0.943 ± 0.564	0.831 ± 0.349	1.137 ± 0.787	<0.001*	<0.001*, ^b^
MMSE	Mean ± SD	26.76 ± 1.11	21.72 ± 4.21	22.84 ± 2.87	19.74 ± 5.38	<0.001*	<0.001*, ^c^

Data are reported for both biomarker‐based (AD+/AD–) and clinical (HC/MCI/ADD) classification, with the AD+ group disclosed in MCI and ADD. Chi‐square test was performed for categorical variables, Mann–Whitney test (AD+ vs AD–) or Kruskal–Wallis test with Mann–Whitney post hoc (HC vs MCI vs ADD) for continuous variables. *APOE* status is reported based on the presence of the ε4 allele (ε4%), in either heterozygous or homozygous form.

Abbreviations: AD, Alzheimer's disease; ADD, Alzheimer's disease dementia; HC, healthy control; MCI, mild cognitive impairment; MMSE, Mini‐Mental State Examination; SD, standard deviation.

^a^ Chi‐square test: ADD vs MCI (*p *= 0.369), MCI vs HC (*p *= 0.012), ADD vs HC (*p *= 0.157).

^b^ Pair‐wise comparisons (Mann–Whitney test): ADD vs MCI (*p *= 0.177), MCI vs HC (*p *< 0.001), ADD vs HC (*p *< 0.001).

^c^ Pair‐wise comparisons (Mann–Whitney test): ADD vs MCI (*p *= 0.117), MCI vs HC (*p *= 0.001), ADD vs HC (*p *< 0.001).

^*^ *p*‐value < 0.05.

### 3.2 LC‐MRI analysis and TAM plasma levels

**FIGURE 1:**
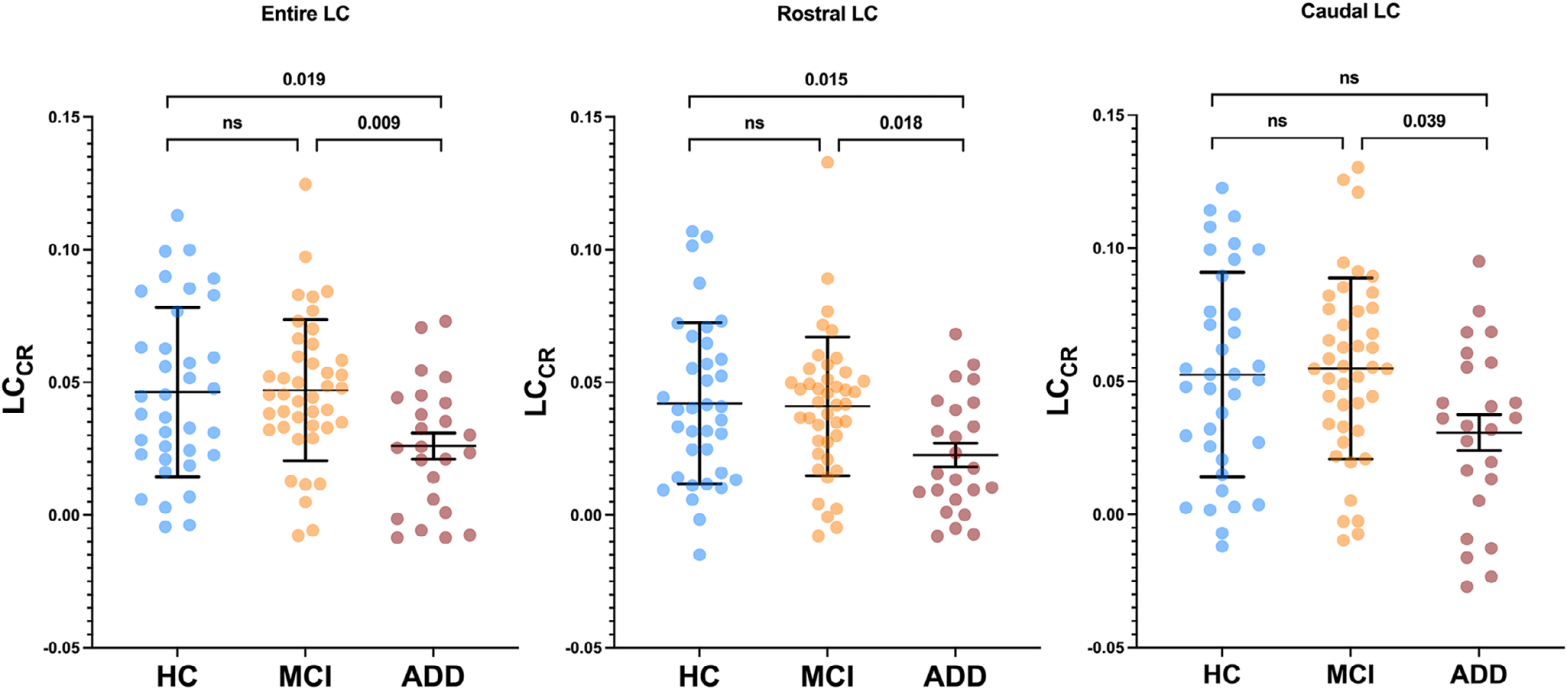
LC_CR_ across diagnostic groups. Scatter plot of LC_CR_ parameter in the three diagnostic groups (HC, MCI, and ADD), considering the entire LC and its two rostral and caudal subregions. Trend lines represent median and interquartile range. Nonparametric tests were used. ADD, Alzheimer's disease dementia; HC, healthy control; LC, locus coeruleus; LC_CR_, locus coeruleus contrast‐ratio; MCI, mild cognitively impaired.

**FIGURE 2:**
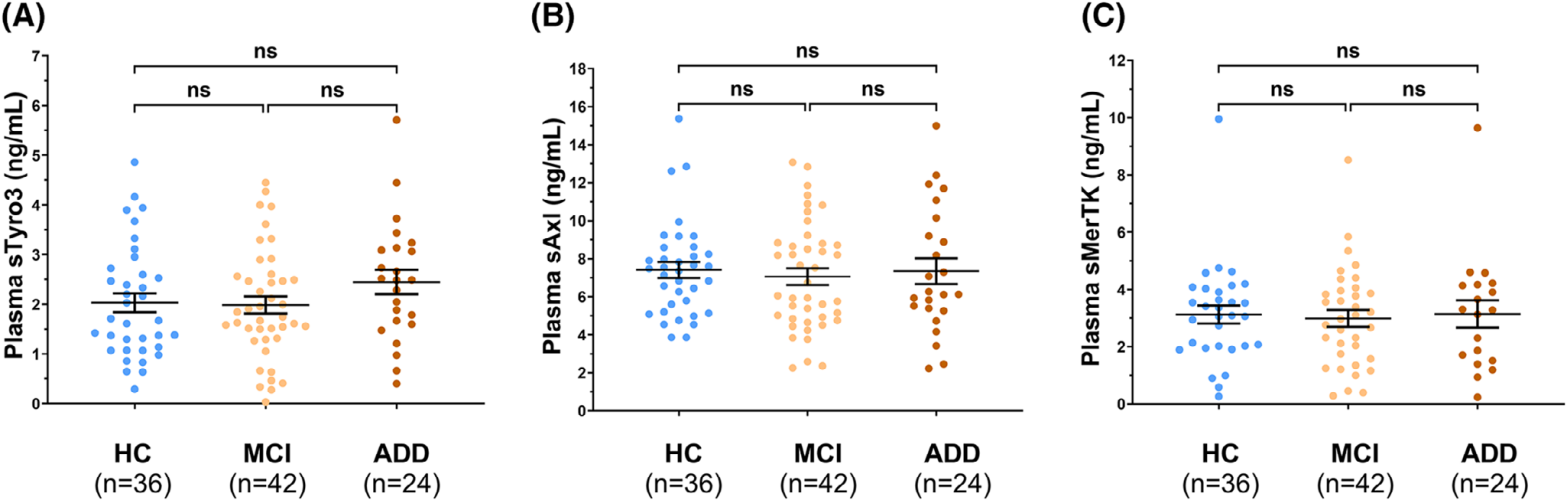
TAM receptors plasma levels. Plots representing the levels of sTyro3 (A), sAxl (B), and sMerTK (B) receptors in the plasma of heathy controls (HCs), mild cognitive impairment (MCI), and Alzheimer's disease dementia (ADD) patients (ng/mL). Data are presented as mean ± SEM. *p* values were calculated using a Kruskal‐Wallis test. ns, non‐significant. sAxl, soluble Axl; sMerTK, soluble MerTK; sTyro3, soluble Tyro3; TAM, Tyro3, Axl, and MerTK.

ADD patients showed significantly lower values of LC_CR_ than HC individuals when considering both the entire LC (*p* = 0.019) and its rostral part (*p* = 0.015) (Figure 1). A similar difference—in this case extending also to the caudal part of the LC—was found when comparing with the MCI group (Figure 1). The latter did not show a significant difference with the HC groups (Figure 1). No significant differences were found among diagnostic groups for the plasmatic levels of soluble TAM receptors (Figure 2).

### 3.3 Association between plasmatic level of TAMs, LC_CR_ and pTau217

**FIGURE 3:**
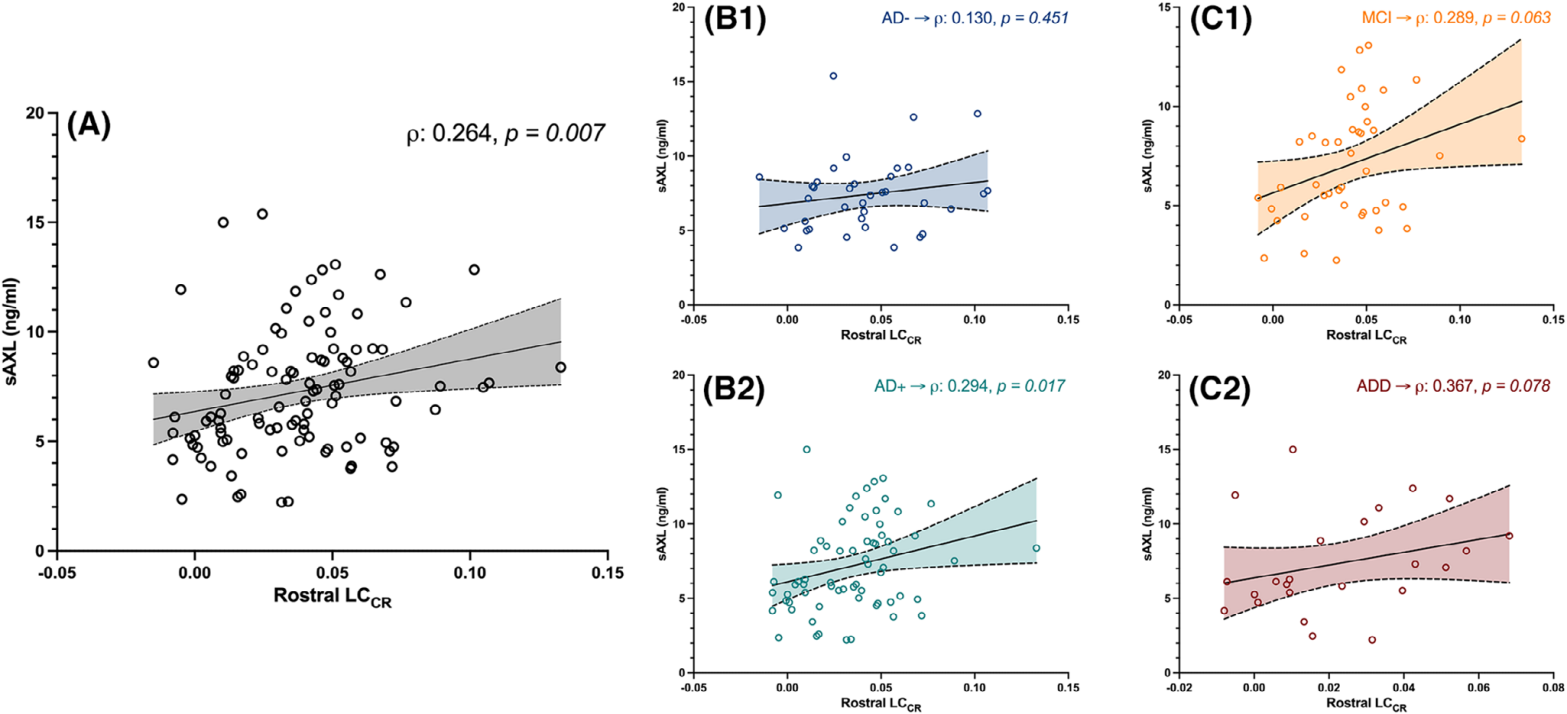
Correlation between LC_CR_ and sAxl levels in AD+ patients. Dot plots report the direct correlation between the integrity of rostral LC and plasmatic level of sAxl receptor, in the whole cohort (A) and when subdividing this latter into AD+/AD‐ (HC) subjects (B1‐2), or into mild cognitive impairment (MCI) and Alzheimer's disease dementia (ADD) patients (C1‐2). ρ and *p values* were obtained using Spearman's correlation test.

A direct correlation was found between rostral LC_CR_ and plasmatic levels of sAxl in the whole cohort (ρ = 0.264, *p* = 0.007). The same association was detected in the AD+ group (ρ = 0.294, *p* = 0.017) but not in the AD− group (*p* = 0.451). The association is strengthened when considering the MCI (ρ = 0.289) and ADD (ρ = 0.367) groups separately, although it lost statistical significance (*p* = 0.063 and *p* = 0.078, respectively) (Figure 3). No other significant associations were found when considering other LC regions or the other TAM receptors.

Plasma levels of p‐tau217 were not significantly associated with LC_CR_, either in the entire nucleus or its subregions, or with plasma TAM levels (*not shown*).

## 4 DISCUSSION

In this study, we aimed to explore the occurrence of an association between LC degeneration in AD and the degree of neuroinflammatory response. We used MRI to evaluate in vivo LC integrity and assessed plasmatic levels of soluble TAM receptors. Here, we report evidence of an association between higher plasma sAxl levels and rostral LC integrity preservation, occurring specifically in patients with AD pathology. To our knowledge, this is the first time that this phenomenon has been revealed in vivo in patients with AD.

Enhanced levels of sTAM have been reported in several autoimmune affections: systemic lupus erythematosus, rheumatoid arthritis, Sjogren's syndrome, or Behcet's disease. Elevated sTAM levels are also found in patients with type 2 diabetic nephropathy and liver cirrhosis.^33^ This may reflect reduced native receptor availability and increased soluble decoys, leading to diminished TAM‐mediated immune suppression.

In neurodegenerative diseases, sAxl has been proposed as a biomarker of multiple sclerosis (MS) disability, with serum levels differing by impairment status and treatment response.^34^ In MS, TAM receptors are implicated in neuronal remyelination,^35^ and Axl loss in mouse models leads to increased demyelination and axonal damage.^36^

After nearly two decades of use,^37^ LC‐MRI has proven reliable in AD research, supported by histological validation,^38^, ^39^ tau pathology correlations,^39^, ^40^ and a growing number of studies in which acquisition and post hoc processing protocols have been validated by many research groups.^7^, ^8^, ^41^, ^42^ Using our validated method,^9^ we observed significant LC signal disruption in AD patients compared to HCs, both considering the whole nucleus and its rostral part, consistently with previous data.^7^, ^9^, ^40^, ^43^ The specific vulnerability of the rostral part of LC has also been observed in other studies.^9^, ^40^, ^43^ A possible explanation has been provided based on the topographical organization of LC‐NA neurons, as those projecting to the limbic structures—primarily affected in AD—are located mainly in the most rostral part of the nucleus.^44^, ^45^

In our opinion, this premise strengthens the neurobiological significance of our findings, as we observed that the association with plasmatic sAxl levels occurred only when considering the rostral part of the LC and not its caudal part or the nucleus as a whole. Furthermore, the LC‐sAxl relation was detected when considering the AD+ patients group, whereas it disappeared in the AD– control groups. This suggests a pathophysiological link driven by AD pathology, supporting two interpretations.

First, Axl, part of the TAM family with Tyro3 and Mer,^46^ is expressed mainly by microglia^47^ and regulates phagocytosis and neuroinflammation.^46^, ^47^, ^48^ It is intriguing that although Mer seems to be crucial in modulating resting microglia, Axl activation and expression are increased mainly in pro‐inflammatory conditions.^46^, ^47^ Its activation supports microglial phagocytosis,^48^ and thus higher activation of Axl might be interpreted as a physiological response to an inflammatory environment, not necessarily acquiring a noxious role.^46^, ^47^ Clinical studies show baseline Axl correlates with cognitive impairment, but also with slower disease progression.^23^, ^24^, ^49^ These findings align with Axl's dual role: upregulated in inflammation, yet beneficial via microglial modulation. NA also regulates microglial activity,^12^, ^49^ and LC‐NA system impairment in AD models disrupts glial phagocytosis and promotes amyloid accumulation.^16^, ^17^ NA modulates microglia activity, both maintaining it in its resting state and promoting its efficient activation in response to pathological event.^50^, ^51^, ^52^ Thus, early LC‐NA dysfunction may exacerbate neuroinflammation in humans.^10^, ^12^, ^49^ Our observed sAxl–LC link may reflect this mechanism: preserved LC‐NA function could enhance TAM signaling and sAxl levels. Notably, sAxl may act as a decoy receptor for Gas6,^53^ but elevated levels could also result from increased Axl expression, not just shedding, thereby preserving TAM signaling. This might be the explanation beyond our observation that higher plasma sAxl levels correlate with preserved LC integrity. Unfortunately, to the best of our knowledge, no experimental or in vitro studies have been performed specifically exploring a possible role of NA in modulating TAM receptor expression and activity, thus our hypothesis cannot rely on further evidence.

Second, TAM activity might protect the LC from tau pathology.^4^, ^5^ AD+ patients with higher sAxl may have more resilient LC structures. Of interest, the LC uniquely expresses the TAM ligand Pros1, although it activates Tyro3 and MerTK, not Axl, which binds Gas6.^54^ However, as Pros1 specifically activates Tyro3 and Mer but not Axl,^55^ which in turns shows higher affinity with Gas6, expressed throughout the entire CNS,^54^, ^55^ we cannot further speculate on this evidence to interpret our results.

Another point to mention is that it is not clear whether all the sAxl in the plasma originates from a CSF spillover. Additional peripheric sources could play a role. Indeed, TAM receptors are not only expressed by central microglia, but also by peripheral macrophages and dendritic cells.^56^

This study has limitations. The absence of amyloid biomarkers prevented assessment of cortical amyloid burden and its relationship with LC‐NA integrity and TAM levels. This also limited diagnostic precision. Nonetheless, our clinical, neurological, and radiological assessments, along with plasma p‐tau217 levels, provide a robust foundation for our conclusions.

The second significant limitation we acknowledge is the relatively small sample size, primarily due to the single‐center design of the study. This limited sample size may have reduced our statistical power, particularly considering the inconsistent findings in the LC caudal region. Although the significant difference between MCI and ADD was expected (consistently with post‐mortem studies^5^), the lack of significance in the comparison between ADD and HC might be attributed to this statistical limitation. The same limit might, at least in part, justify the lack of significant differences between HC and MCI patients in the LC‐MRI analysis and the absence of differences in plasma TAM receptor levels across the three diagnostic groups. However, the absence of longitudinal data, which in other studies has provided greater insight into these parameters, may also have contributed to this negative finding.^9^, ^23^, ^24^, ^25^

Nonetheless, despite these limitations, we believe our findings warrant attention due to their novelty and potential pathophysiological significance. As noted, this is the first in vivo study to highlight a possible association between LC degeneration and AD‐related neuroinflammation in patients. In addition, we provide preliminary clinical evidence suggesting a potential interplay between the TAM receptor system and NA, which has never been explored experimentally.

For these reasons, further research is necessary. Our findings should be tested and replicated in larger, longitudinal clinical studies with an exhaustive assessment of AD biomarkers. Experimental studies, both in vitro and in animal models, could further investigate a possible interaction between TAM receptors and NA signaling in microglial cells. This could provide new insights into the role of neuroinflammation in AD and potentially uncover novel therapeutic targets for disease‐modifying interventions.

## CONFLICT OF INTEREST STATEMENT

The authors report no competing interests.

## ETHICS STATEMENT

The human work has been authorized by the Ethics Committee of Area Vasta Nord‐Ovest of Tuscany Region Health System, (#1203 Protocol PE‐2013‐02359574), and approved by the Ethics Review Panel of the University of Luxembourg (Project ERP 23‐050 HEN1_COLAB_Giorgi).

## Supporting information



Supporting Information

Supporting Information

## Data Availability

The data supporting the findings of this study are not publicly available due to the potential presence of information that could compromise the privacy of research participants but are available from F.S.G. (filippo.giorgi@unipi.it) upon reasonable request.

## References

[1] Counts SE , Mufson EJ . Chapter 12-Locus coeruleus. In The Human Nervous System. 3rd ed.; Mai JK, Paxinos, G, Eds; Academic Press: San Diego, CA, USA, 2012:425‐438. doi:10.1016/B978-0-12-374236-0.10012-4

[2] Poe GR , Foote S , Eschenko O , et al. Locus coeruleus: a new look at the blue spot. Nat Rev Neurosci. 2020;21: 644‐659. doi:10.1038/s41583-020-0360-9 32943779 PMC8991985

[3] Braak H , Thal DR , Ghebremedhin E , Del Tredici K . Stages of the pathologic process in Alzheimer disease: age categories from 1 to 100 years. J Neuropathol Exp Neurol. 2011;70: 960‐969. doi:10.1097/NEN.0b013e318232a379 22002422

[4] Theofilas P , Dunlop S , Heinsen H , Grinberg LT . Turning on the light within: subcortical nuclei of the isodentritic core and their role in Alzheimer's disease pathogenesis. J Alzheimers Dis. 2015;46: 17‐34. doi:10.3233/JAD-142682 25720408 PMC4550582

[5] Theofilas P , Ehrenberg AJ , Dunlop S , et al. Locus coeruleus volume and cell population changes during Alzheimer's disease progression: a stereological study in human postmortem brains with potential implication for early‐stage biomarker discovery. Alzheimers Dement. 2017;13: 236‐246. doi:10.1016/j.jalz.2016.06.2362 27513978 PMC5298942

[6] Kelly SC , He B , Perez SE , Ginsberg SD , Mufson EJ , Counts SE . Locus coeruleus cellular and molecular pathology during the progression of Alzheimer's disease. Acta Neuropathol Commun. 2017;5: 8. doi:10.1186/s40478-017-0411-2 28109312 PMC5251221

[7] Jacobs HIL , Becker JA , Kwong K , et al. In vivo and neuropathology data support locus coeruleus integrity as indicator of Alzheimer's disease pathology and cognitive decline. Sci Transl Med. 2021;13: eabj2511. doi:10.1126/scitranslmed.abj2511 34550726 PMC8641759

[8] Dahl MJ , Mather M , Werkle‐Bergner M , et al. Locus coeruleus integrity is related to tau burden and memory loss in autosomal‐dominant Alzheimer's disease. Neurobiol Aging. 2022;112: 39‐54. doi:10.1016/j.neurobiolaging.2021.11.006 35045380 PMC8976827

[9] Galgani A , Lombardo F , Martini N , et al. Magnetic resonance imaging Locus Coeruleus abnormality in amnestic Mild Cognitive Impairment is associated with future progression to dementia. Eur J Neurol. 2023;30: 32‐46. doi:10.1111/ene.15556 36086917 PMC10092028

[10] Heneka MT , Carson MJ , El Khoury J , et al. Neuroinflammation in Alzheimer's disease. Lancet Neurol. 2015;14: 388‐405. doi:10.1016/S1474-4422(15)70016-5 25792098 PMC5909703

[11] Heneka MT , O'Banion MK , Terwel D , Kummer MP . Neuroinflammatory processes in Alzheimer's disease. J Neural Transm (Vienna). 2010;117: 919‐947. doi:10.1007/s00702-010-0438-z 20632195

[12] Giorgi FS , Saccaro LF , Galgani A , et al. The role of Locus Coeruleus in neuroinflammation occurring in Alzheimer's disease. Brain Res Bull. 2019;153: 47‐58. doi:10.1016/j.brainresbull.2019.08.007 31419539

[13] Heneka MT , Nadrigny F , Regen T , et al. Locus ceruleus controls Alzheimer's disease pathology by modulating microglial functions through norepinephrine. Proc Natl Acad Sci U S A. 2010;107: 6058‐6063. doi:10.1073/pnas.0909586107 20231476 PMC2851853

[14] Heneka MT , Gavrilyuk V , Landreth GE , O'Banion MK , Weinberg G , Feinstein DL . Noradrenergic depletion increases inflammatory responses in brain: effects on IkappaB and HSP70 expression. J Neurochem. 2003;85: 387‐398. doi:10.1046/j.1471-4159.2003.01694.x 12675915

[15] Jardanhazi‐Kurutz D , Kummer MP , Terwel D , Vogel K , Thiele A , Heneka MT . Distinct adrenergic system changes and neuroinflammation in response to induced locus ceruleus degeneration in APP/PS1 transgenic mice. Neuroscience. 2011;176: 396‐407. doi:10.1016/j.neuroscience.2010.11.052 21129451

[16] Heneka MT , Ramanathan M , Jacobs AH , et al. Locus ceruleus degeneration promotes Alzheimer pathogenesis in amyloid precursor protein 23 transgenic mice. J Neurosci. 2006;26: 1343‐1354. doi:10.1523/JNEUROSCI.4236-05.2006 16452658 PMC6675491

[17] Jardanhazi‐Kurutz D , Kummer MP , Terwel D , et al. Induced LC degeneration in APP/PS1 transgenic mice accelerates early cerebral amyloidosis and cognitive deficits. Neurochem Int. 2010;57: 375‐382. doi:10.1016/j.neuint.2010.02.001 20144675

[18] Dahl MJ , Bachman SL , Dutt S , et al. The integrity of dopaminergic and noradrenergic brain regions is associated with different aspects of late‐life memory performance. Nat Aging. 2023;3: 1128‐1143. doi:10.1038/s43587-023-00469-z 37653256 PMC10501910

[19] Zhou S , Li Y , Zhang Z , Yuan Y . An insight into the TAM system in Alzheimer's disease. Int Immunopharmacol. 2023;116: 109791. doi:10.1016/j.intimp.2023.109791 36738678

[20] Mattsson N , Insel P , Nosheny R , et al. CSF protein biomarkers predicting longitudinal reduction of CSF β‐amyloid42 in cognitively healthy elders. Transl Psychiatry. 2013;3: e293. doi:10.1038/tp.2013.69 23962923 PMC3756294

[21] Theeke LA , Liu Y , Wang S , et al. Plasma proteomic biomarkers in Alzheimer's disease and cardiovascular disease: a longitudinal study. Int J Mol Sci. 2024;25: 10751. doi:10.3390/ijms251910751 39409080 PMC11477191

[22] Pereira JB , Janelidze S , Strandberg O , et al. Microglial activation protects against accumulation of tau aggregates in nondemented individuals with underlying Alzheimer's disease pathology. Nat Aging. 2022;2: 1138‐1144. doi:10.1038/s43587-022-00310-z 37118533 PMC10154192

[23] Brosseron F , Maass A , Kleineidam L , et al. Soluble TAM receptors sAXL and sTyro3 predict structural and functional protection in Alzheimer's disease. Neuron. 2022;110: 1009‐1022.e4. doi:10.1016/j.neuron.2021.12.016 34995486

[24] Hayek D , Ziegler G , Kleineidam L , et al. Different inflammatory signatures based on CSF biomarkers relate to preserved or diminished brain structure and cognition. Mol Psychiatry. 2024;29: 992‐1004. doi:10.1038/s41380-023-02387-3 38216727 PMC11176056

[25] Brosseron F , Maass A , Kleineidam L , et al. Serum IL‐6, sAXL, and YKL‐40 as systemic correlates of reduced brain structure and function in Alzheimer's disease: results from the DELCODE study. Alzheimers Res Ther. 2023;15: 13. doi:10.1186/s13195-022-01118-0 36631909 PMC9835320

[26] McKhann GM , Knopman DS , Chertkow H , et al. The diagnosis of dementia due to Alzheimer's disease: recommendations from the National Institute on Aging‐Alzheimer's Association workgroups on diagnostic guidelines for Alzheimer's disease. Alzheimers Dement. 2011;7: 263‐269. doi:10.1016/j.jalz.2011.03.005 21514250 PMC3312024

[27] Albert MS , DeKosky ST , Dickson D , et al. The diagnosis of mild cognitive impairment due to Alzheimer's disease: recommendations from the National Institute on Aging‐Alzheimer's Association workgroups on diagnostic guidelines for Alzheimer's disease. Alzheimers Dement. 2011;7: 270‐279. doi:10.1016/j.jalz.2011.03.008 21514249 PMC3312027

[28] Folstein MF , Folstein SE , McHugh PR . “Mini‐mental state”. A practical method for grading the cognitive state of patients for the clinician. J Psychiatr Res. 1975;12: 189‐198. doi:10.1016/0022-3956(75)90026-6 1202204

[29] Hughes CP , Berg L , Danziger WL , Coben LA , Martin RL . A new clinical scale for the staging of dementia. Br J Psychiatry. 1982;140: 566‐572. doi:10.1192/bjp.140.6.566 7104545

[30] Cummings JL , Mega M , Gray K , Rosenberg‐Thompson S , Carusi DA , Gornbein J . The Neuropsychiatric Inventory: comprehensive assessment of psychopathology in dementia. Neurology. 1994;44: 2308‐2314. doi:10.1212/wnl.44.12.2308 7991117

[31] Mammel AE , Hsiung G‐YR , Mousavi A , et al. Clinical decision points for two plasma p‐tau217 laboratory developed tests in neuropathology confirmed samples. Alzheimer Dement. 2025;17: e70070. doi:10.1002/dad2.70070 PMC11736701 39822298

[32] Liu C‐C , Liu C‐C , Kanekiyo T , Xu H , Bu G . Apolipoprotein E and Alzheimer disease: risk, mechanisms and therapy. Nat Rev Neurol. 2013;9: 106‐118. doi:10.1038/nrneurol.2012.263 23296339 PMC3726719

[33] Malikova I , Worth A , Aliyeva D , Khassenova M , Kriajevska MV , Tulchinsky E . Proteolysis of TAM receptors in autoimmune diseases and cancer: what does it say to us?. Cell Death Dis. 2025;16: 1‐13. doi:10.1038/s41419-025-07480-9 40044635 PMC11883011

[34] D'Onghia D , Colangelo D , Bellan M , et al. Gas6/TAM system as potential biomarker for multiple sclerosis prognosis. Front Immunol. 2024;15: 1362960. doi:10.3389/fimmu.2024.1362960 38745659 PMC11091300

[35] Rosenstein I , Novakova L , Kvartsberg H , et al. Tyro3 and Gas6 are associated with white matter and myelin integrity in multiple sclerosis. J Neuroinflamm. 2024;21: 320. doi:10.1186/s12974-024-03315-0 PMC11645787 39673059

[36] Weinger JG , Brosnan CF , Loudig O , et al. Loss of the receptor tyrosine kinase Axl leads to enhanced inflammation in the CNS and delayed removal of myelin debris during experimental autoimmune encephalomyelitis. J Neuroinflammation. 2011;8: 49. doi:10.1186/1742-2094-8-49 21569627 PMC3121615

[37] Shibata E , Sasaki M , Tohyama K , et al. Age‐related changes in locus ceruleus on neuromelanin magnetic resonance imaging at 3 Tesla. Magn Reson Med Sci. 2006;5: 197‐200. doi:10.2463/mrms.5.197 17332710

[38] Keren NI , Taheri S , Vazey EM , et al. Histologic validation of locus coeruleus MRI contrast in post‐mortem tissue. Neuroimage. 2015;113: 235‐245. doi:10.1016/j.neuroimage.2015.03.020 25791783 PMC4649944

[39] Hary AT , Chadha S , Mercaldo N , et al. Locus coeruleus tau validates and informs high‐resolution MRI in aging and at earliest Alzheimer's pathology stages. Acta Neuropathol Commun. 2025;13: 44. doi:10.1186/s40478-025-01957-6 40022196 PMC11871710

[40] Van Egroo M , Riphagen JM , Ashton NJ , et al. Ultra‐high field imaging, plasma markers and autopsy data uncover a specific rostral locus coeruleus vulnerability to hyperphosphorylated tau. Mol Psychiatry. 2023;28: 2412‐2422. doi:10.1038/s41380-023-02041-y 37020050 PMC10073793

[41] Giorgi FS , Martini N , Lombardo F , et al. Locus Coeruleus magnetic resonance imaging: a comparison between native‐space and template‐space approach. J Neural Transm (Vienna). 2022;129: 387‐94. doi:10.1007/s00702-022-02486-5 35306617 PMC9007774

[42] Trujillo P , Aumann MA , Claassen DO . Neuromelanin‐sensitive MRI as a promising biomarker of catecholamine function. Brain. 2024;147: 337‐351. doi:10.1093/brain/awad300 37669320 PMC10834262

[43] Dahl MJ , Mather M , Düzel S , et al. Rostral locus coeruleus integrity is associated with better memory performance in older adults. Nat Hum Behav. 2019;3: 1203‐1214. doi:10.1038/s41562-019-0715-2 31501542 PMC7203800

[44] Schwarz LA , Luo L . Organization of the locus coeruleus‐norepinephrine system. Curr Biol. 2015;25: R1051‐6. doi:10.1016/j.cub.2015.09.039 26528750

[45] Szabadi E . Functional neuroanatomy of the central noradrenergic system. J Psychopharmacol. 2013;27: 659‐693. doi:10.1177/0269881113490326 23761387

[46] Tondo G , Perani D , Comi C . TAM Receptor Pathways at the Crossroads of Neuroinflammation and Neurodegeneration. Dis Markers. 2019;2019: 2387614. doi:10.1155/2019/2387614 31636733 PMC6766163

[47] Fourgeaud L , Través PG , Tufail Y , et al. TAM receptors regulate multiple features of microglial physiology. Nature. 2016;532: 240‐244. doi:10.1038/nature17630 27049947 PMC5358512

[48] Repici A , Ardizzone A , De Luca F , et al. Signaling pathways of AXL receptor tyrosine kinase contribute to the pathogenetic mechanisms of glioblastoma. Cells. 2024;13: 361. doi:10.3390/cells13040361 38391974 PMC10886920

[49] Feinstein DL , Kalinin S , Braun D . Causes, consequences, and cures for neuroinflammation mediated via the locus coeruleus: noradrenergic signaling system. J Neurochem. 2016;139(Suppl 2): 154‐178. doi:10.1111/jnc.13447 26968403

[50] Sugama S , Kakinuma Y . Noradrenaline as a key neurotransmitter in modulating microglial activation in stress response. Neurochem Int. 2021;143: 104943. doi:10.1016/j.neuint.2020.104943 33340593

[51] Braun D , Madrigal JLM , Feinstein DL . Noradrenergic regulation of glial activation: molecular mechanisms and therapeutic implications. Curr Neuropharmacol. 2014;12: 342‐352. doi:10.2174/1570159X12666140828220938 25342942 PMC4207074

[52] Gyoneva S , Traynelis SF . Norepinephrine modulates the motility of resting and activated microglia via different adrenergic receptors. J Biol Chem. 2013;288: 15291‐15302. doi:10.1074/jbc.M113.458901 23548902 PMC3663549

[53] Kariolis MS , Miao YR , Jones DS , et al. An engineered Axl “decoy receptor” effectively silences the Gas6‐Axl signaling axis. Nat Chem Biol. 2014;10: 977‐983. doi:10.1038/nchembio.1636 25242553 PMC4372605

[54] Prieto AL , Weber JL , Tracy S , Heeb MJ , Lai C . Gas6, a ligand for the receptor protein‐tyrosine kinase Tyro‐3, is widely expressed in the central nervous system. Brain Res. 1999;816: 646‐661. doi:10.1016/s0006-8993(98)01159-7 9878891

[55] Lew ED , Oh J , Burrola PG , et al. Differential TAM receptor‐ligand‐phospholipid interactions delimit differential TAM bioactivities. Elife. 2014;3: e03385. doi:10.7554/eLife.03385 25265470 PMC4206827

[56] Prouse T , Majumder S , Majumder R . Functions of TAM receptors and ligands protein S and Gas6 in atherosclerosis and cardiovascular disease. Int J Mol Sci. 2024;25: 12736. doi:10.3390/ijms252312736 39684449 PMC11641688

