# Supplementary material for: Locus coeruleus integrity correlates with plasma soluble Axl levels in Alzheimer's disease patients
Source: Alzheimers Dement. 2025 Jul 1;21(7):e70434. doi: 10.1002/alz.70434 (PMC12213453; doi:10.1002/alz.70434)
Supplement: Supplementary file 2 — Supporting Information [file ALZ-21-e70434-s001.docx]

**Supplementary Methods**

**1. LC-MRI Protocol**

All participants underwent High-Field 3T Brain MRI within one month of neurological assessment. Imaging was performed using a General Electric Excite HDx scanner equipped with an eight-channel phased-array head coil. The protocol included 2D fluid-attenuated inversion recovery (FLAIR), T2*-weighted gradient recalled echo, spin-echo T1-weighted, fat-saturated fast spin-echo (FSE) T2-weighted, diffusion-weighted imaging (DWI), and 3D fast spoiled gradient recalled (FSPGR) T1-weighted sequences for comprehensive whole-brain assessment.

For LC imaging, we employed a 2D FSE T1-weighted sequence with the following parameters: repetition time (TR) = 600 ms, echo time (TE) = 14 ms, flip angle = 90°, echo train length = 2, number of excitations (NEX) = 5, matrix size = 512 × 384, field of view (FOV) = 200 × 200 mm, in-plane resolution = 0.39 × 0.52 mm, slice thickness = 2.2 mm, no interslice gap, and acquisition time = 14.29 minutes. The acquisition plane was oblique axial, oriented perpendicular to the floor of the fourth ventricle, spanning from the inferior pons to the posterior commissure, ensuring full rostrocaudal coverage of the LC in all subjects.

**1.1 Template Construction**

Study-specific templates were generated for both 3D anatomical and LC-targeted MRI scans using the method described by Dahl et al. [1], with minor changes, as outlined below. The template was developed in the context of a previous study [2] and is based on the MRI scans of the included participants.

*1.1.1 3D Anatomical Template*

A common anatomical space (template3D) was created using only the 3D T1-weighted images of cognitively healthy control subjects. This was achieved through multiresolution, iterative group-wise registration using antsMultivariateTemplateConstruction2 from Advanced Normalization Tools (ANTs) v2.3.4 [3]. Prior to registration, each scan was resampled to an isotropic resolution of 0.5 mm, and non-uniform intensity bias was corrected using N4 bias field correction. Registration parameters included: 30x90x20 iterations across resolution levels, cross-correlation as the similarity metric, and the Greedy SyN transformation model. The resulting aligned volumes were averaged to produce the template3D. Corresponding transformation matrices and deformation fields mapping each subject’s native space to the template space were also preserved.

*1.1.2 Brainstem Template for LC Imaging*

To generate the brainstem template from LC-targeted scans, individual LC-MRI images were first warped into the 3D template space using the transformation fields from the prior step. These resampled images were then subjected to a second group-wise registration using the same parameters, producing a high-resolution (0.5 mm isotropic) brainstem template that accounted for both inter-individual anatomical variability and potential intra-subject misalignment between 3D and LC-targeted scans.

*1.1.3 LC Mask Creation*

The brainstem template demonstrated a favorable signal-to-noise ratio and showed marked hyperintensities in bilateral regions beneath the floor of the fourth ventricle—consistent with LC anatomy. A semi-automated threshold-based segmentation approach was employed to derive the LC mask. Using ITK-SNAP [4], an experienced operator placed bilateral square ROIs (8.5 mm × 8.5 mm) in the ventral pons across slices. For each hemisphere, a custom Python script identified voxels exceeding the threshold of μROI + 4σROI, where μROI and σROI represent the mean and standard deviation of signal intensity within the reference ROIs. The resulting candidate LC voxels were manually refined to exclude anatomically implausible regions (e.g., ventricular space). The finalized mask comprised 17 slices and included 234 voxels.

**1.2 Extraction of LC MRI Parameters**

The templates described above were constructed using data from cognitively healthy controls only. For analysis, LC-targeted images from all participants (including patients and controls) were registered to the brainstem template space.

The parameter LC_CR_, i.e. the contrast-ratio of the LC, was calculated using the following formula:

**LC_CR_ = [max(LC) - max(Ref)] / max(Ref)**

where max(Ref) and max(LC) are the maximum signal intensities in the left and right reference regions, and in LC regions derived from the LC template segmentation, respectively.

To assess regional variations along the rostrocaudal axis of the LC, the full LC mask was divided into two subregions (rostral and caudal), each comprising 9 slices with one-slice overlap. For each hemisphere, LC_CR_ values were averaged within each subregion and across the entire LC. Both left and right LC values were computed and averaged for subsequent statistical analysis

**References to Supplementary Methods**

1. Dahl MJ, Mather M, Düzel S, et al. Rostral locus coeruleus integrity is associated with better memory performance in older adults. Nature Human Behaviour 2019;3(11):1203–1214. doi:10.1038/s41562-019-0715-2
2. Galgani A, Lombardo F, Martini N, et al. Magnetic resonance imaging Locus Coeruleus abnormality in amnestic Mild Cognitive Impairment is associated with future progression to dementia. *Eur J Neurol*. 2023;30(1):32-46. doi:10.1111/ene.15556
3. Avants BB, Tustison NJ, Song G, et al. A reproducible evaluation of ANTs similarity metric performance in brain image registration. NeuroImage 2011;54(3):2033–2044. doi:10.1016/j.neuroimage.2010.09.025.
4. Yushkevich PA, Piven J, Hazlett HC, Gimpel Smith R, Ho S, Gee JC, and Gerig G. User-guided 3D active contour segmentation of anatomical structures: Significantly improved efficiency and reliability. Neuroimage 2006 Jul 1;31(3):1116-28.
